# Tri-State Medical Association

**Published:** 1890-12

**Authors:** 


### Society Reports.

### TRI-STATE MEDICAL ASSOCIATION.

*Second Annual Meeting, Held in Chattanooga, Tennessee, October 14, 15 and 16, 1890.*

### OCTOBER 14TH—FIRST DAY—MORNING SESSION.

The Association met in Turner Hall, and was called to order by the President, Dr. J. B. Cowan, of Tullahoma, Tennessee, at 10 A. M.

Prayer was offered by the Rev. Charles Hyde.

The remainder of the first session was devoted to introductions, miscellaneous business, etc.

### FIRST DAY—AFTERNOON SESSION.

Dr. W. P. McDonald, of Hill City, Tennessee, contributed a paper entitled

### REPORT OF A CASE OF CANCRUM ORIS OR GANGRENOUS STOMATITIS.

The patient, white, was four years of age. Dr. McDonald first saw the case on August 5th. He found her with some fever, tongue coated brown, with red edges, surface more or less furrowed or full of cracks in the brown coat, her general appearance indicating a very low state of health. Her bowels were inclined to be too loose, and her abdomen seemed to be somewhat distended—tympanitic. With these symptoms he pronounced the case one of typho-malarial fever. He began treatment by giving a mercurial purge, followed by large doses of quinine and bismuth. This treatment was continued for two days, after which he ordered ten drops of the syrup of iodide of iron after meals, also quinine and bismuth in small doses three or four times a day. This treatment was continued for about ten days, at the end of which time he found the patient had greatly improved.

Fourteen days after seeing the child for the first time, he was called to see the mother, whom he found with nearly the same symptoms as the child first presented. During his absence from the city, another physician had treated an older daughter, whom he supposed had been troubled with the same disease. The child was still improving, except complaining of a sore throat. On examination he found several small ulcers on the right side of the mouth, with a general inflammatory appearance of the gums and whole mucous lining of that side of the buccal cavity, with some bleeding from around the teeth. The trouble at first seemed to yield to a wash of chlorate of potassium and creosote, but on the 17th day of illness the inflammation increased rapidly, the whole cheek and side of the face appearing very much swollen, and the inside was fast becoming dark and gangrenous. August 24th a small dark spot about the size of a a penny made its appearance externally just at the right wing of the nose. This rapidly enlarged, involving the wing of the nose and in proportion the tissues on either side of the central spot; and on August 25th it had involved the right side of the nose up to the inner canthus of the eye, also the upper lip to the median line, and had spread rapidly on the cheek, reaching a point where the zygomatic muscles cross the superficial portion of the masseter. The teeth on this side became loose and dropped out, both above and below, indicating deep-seated trouble, possibly involving the maxillary bone.

Careful investigation revealed a strumous diathesis existing in the family, but no history of tuberculosis could be ascertained.

According to good authorities cancrum oris more frequently occurs as a sequel of other diseases than *per se*. The statistics of Rilliet and Barthez show that out of 98 cases of this disease, 41 were following measles; 5 scarlet fever; 6 whooping cough;

9 intermittent fever; 9 typhoid fever, and 7 mercurial salivation.

To draw a conclusion in the case by these statistics and by watching the case closely from the start, Dr. McDonald asks, "Did this occur as the sequal of the typho-malarial fever which I diagnosed (remembering the poor state of health the child was in to begin with)? Or was it the result of mercurial salivation produced by the mercury given at the beginning and followed two days after by the syrup of the iodide of iron?"

Dr. James E. Reeves, of Chattanooga, said the case was evidently one of gangrene, which is almost necessarily fatal.

President Cowan asked if the character of the disease might not have changed. To which Dr. Reeves replied that there was nothing impossible in medicine, and the disease might have changed.

Dr. E. T. Camp, of Gadsen, Alabama, once saw a case similar to the one reported, which he pronounced gangrena oris, the whole of one cheek being destroyed. There was evidence of mercury having been taken in the early part of the illness of the patient. The case terminated fatally within a few days. The patient was four or five years old and of the lower class. He thought the grangene was the result of mercurial poisoning; mercury was administered during the early part of the illness.

Dr. James E. Reeves, of Chattanooga, then contributed a paper entitled

ON ALL SIDES A LEARNED DOCTOR.

The author introduced his subject by an earnest plea of higher medical education, stigmatizing cheap medical colleges, with no facilities for imparting instruction, as the greatest stumbling-blocks in the way of true progress.

He next paid his attention to the present tendency to make one-sided physicians, and lamented that the department of gynecology was overrun with cheap performers with the speculum, probe, dull curette, etc., and maintained that the specialist should build on the broad foundation of the general practitioner in order to reach professional eminence.

FIRST DAY—EVENING SESSION.

Dr. Andrew Boyd, of Scottsboro, Alabama, read a paper on

FRACTURE AT ELBOW JOINT, and reported cases.

He said after reduction there are but two methods for treatment of fracture at the elbow joint, viz., the extended or straight, and the flexed position. The author thinks the flexed position the better of the two, for the reason, 1, that in all cases surgeons fear anchylosis, and it is much better to have a flexed anchylosed arm than a straight one. The comparative use in each is apparent. 2. When splints have remained twenty-five to thirty days, the arm is atrophied and almost helpless; it is therefore easier to overcome the flexor muscles than the extensors, and a patient can extend an arm with more ease than he can flex it.

Mr. Sydney B. Wright, of Chattanooga, read a paper on

EXPERT TESTIMONY.

He drew the following conclusions:

1. That all the facts proved should be consistent with the hypothesis.

2. That the circumstances should be of a conclusive nature and tendency.

3. That the circumstances should to a moral certainly actually exclude every hypothesis but the one propounded to be proved.

4. That mere circumstantial evidence, unless the chain of circumstances is absolutely complete, ought in no case to be relied upon where direct and positive evidence which might have been given is withheld by the adverse party.

Dr. W. G. Bogart, of Chattanooga, reported

A CASE OF NEUROSIS

in a female, aged seventeen. Examination revealed a coated tongue with a yellowish fur; fœtid breath; temperature 103; pulse 115; bowels constipated, and patient nervous. She had been growing more irritable, less communicative, suffering from sleeplessness, pain in the back of the head and a general depressed feeling for several months. She was placed under treatment and seemed to get along nicely. Six months later the patient was seized with convulsions and was treated therefor. Later she suffered from amenorrhœa.

An interesting feature in the case is that the patient passed three different and distinct kinds of worms. A small black-headed worm, one-half inch long, which had a decided vermicular movement, another a pinheaded worm, and a third a small thread-like worm two inches in length.

In the treatment Dr. Bogart resorted to nearly all the ordinary means, using the bromides, nerve tonics, antispasmodics, anti-periodics, anthelmintics, etc. At present she is taking a uterine tonic combined with a nerve tonic—avena sativa and the fluid extract of aletris. He uses the bromides with chloral at night to produce rest. The patient is also directed to take plenty of outdoor exercise and is thoroughly rubbed night and morning over the spine. She is strictly prohibited from reading books or having them read to her, as she is exceedingly fond of literature.

Dr. W. S. Gahagan, of Chattanooga, read a paper (by title) on Neuralgia.

---

OCTOBER 15TH—SECOND DAY—MORNING SESSION.

The Association called to order by the President at 9 A. M.

The first paper read was by Dr. J. C. Shepard, of Winchester, Tennessee, entitled

A FEW REMARKS ON THE FEVERS OF MIDDLE TENNESSEE AND THEIR TREATMENT.

He said the great malarial period extended from the settlement of the country up to about 1840, during which all the fevers of the country were malarial and periodical. Commencing about 1840, the great typhoid period extended until near 1860. During this period malarial fevers were almost, if not entirely, unknown, and typhoid was dominant everywhere and every case was typical. About 1860, or a little sooner, there was a return of malarial fever, but in connection with typhoid fever. This was the typho-malarial period, which continued for fifteen or twenty years.

About 1880, or somewhat earlier, the characteristic symptoms of both typhoid and malarial fever commenced to disappear and have continued until now. This is the period of fusion. We now have only one fever, which is a continued fever, not typically typhoid, nor malarial, nor even typically typho-malarial. There is not now, nor never was, a continued malarial fever, *per se* in Middle Tennessee, said the speaker.

Of treatment but little is to be said. Cold water baths Dr. Shepard considers impracticable. New antipyretics should be given with caution. He believed that there was entirely too much quinine used in the continued fevers of Middle Tennessee. Physicians could not afford to dispense with alcoholics yet.

Dr. L. P. Barber, of Tracy City, Tennessee, followed with a paper entitled

A CONTRIBUTION TO THE STUDY OF THE CONTINUED FEVERS OF THE SOUTH, in which he said the continued fevers of the South, their nosology and etiology form a subject now justly attracting much attention, a subject upon which much is yet to be learned, and over which the medical world is considerably at variance. Only a close and accurate study of the disease by competent observers, in many and different localities, and a thoughtful comparison of these observations, with free discussion, will advance our knowledge of its nature, and shed light on the vexed question of its cause.

From the first days of his practice, this disease, so common to all parts of Tennessee and her adjoining States, had proved of great interest to him. Encouraged by the recent vigorous inquiry and research in this direction, he began some fifteen months since to keep a record of all fevers that occurred in his private practice, and the comparison of the cases, irrespective of

4

their different designations, had helped him toward a decision as to the nosology of continued fever.

Dr. Barber then reported a large number of interesting cases, after which the two papers were discussed conjointly.

Dr. G. W. Drake, of Chattanooga, in opening the discussion, said that the human body is an aggregation of tissue cells, and that there are found among these cells, in various localities or tissues, certain loose cells, migratory, amœboid, which he would call the police force intended to protect the tissues against the invasion of foreigners. When the foreigner which produces typhoid fever attacks Peyer's patches and the solitary glands, the migratory cells from all adjacent parts, and possibly distant, rush to the conflict. The result is great destruction of life of both microbes and phagocytes, and their putrefying remains produce typhotoxine and probably other alkaloids.

He believed typhoid fever to be produced by a germ once external to the body, but said a "judicious skepticism" was allowable as to whether Eberth's bacillus was the *sole* cause and absolutely necessary to be present in all cases.

Dr. James E. Reeves said it was undoubtedly a fact that the malarial influence was slowly but surely traveling northward.

Dr. J. A. Long, of Long's Mills, Tennessee, related his experience in McMinn county, Tenn. The fevers there had all the symptoms of typhoid fever. There were many non-typical cases. Some were cases of typhoid in a malarial diathesis. He believed that some cases of malarial fever continued because quinine was given in too small doses.

Dr. H. Berlin, of Chattanooga, presented some micro-photographs as an evidence of the existence of microbes in these fevers.

Dr. Geo. A. Baxter, of Chattanooga, suggested the use of salol and naphthol in their treatment.

Dr. J. B. Murfree, of Murfreesboro, Tennessee, thought it was impossible to have two fevers at once. He said there was no typho-malarial fever. We have mild cases of typhoid as well as of other fevers. The use of antiseptics is the proper method of treating typhoid, together with proper nutrition and stimulation.

Dr. Reeves asked Dr. Murfree if he thought typhoid was contagious. Dr. Murfree said it was to a certain extent.

Dr. P. D. Sims, of Chattanooga: "Our fevers are not all either malarial or typhoid. We have another fever dependent upon filth. It may be called *sewage* or *drainage* fever. It is adynamic in type and is liable to take on most of the symptoms of specific typhoid fever. This is the fever now upon us, arising from the continued and increasing pollution of our water supply from sewage sources."

Dr. T. Y. Park, of Peavine, Georgia, suggested that as these fevers presented the symptoms of both fevers, it was practical to use the term typho-malarial, as we cannot make the public understand the technical points of difference and we cannot examine our cases for the micro-organisms.

Dr. Geo. A. Baxter, of Chattanooga, presented a paper on

SILICATE OF SODA—SOME NEW METHODS OF USE IN SURGERY.

The paper chiefly had reference to a silicate jacket made by a new process of hardening the silicate, which, it is claimed, is an improvement on all other jackets inclusive of the plaster of Paris, woven wire, or watch spring now in use for the treatment of spinal injuries or disease. It is lighter, equally durable, equally immobile when on, and capable of removal at any time, and of adjustment to any lateral pressure desired.

### SECOND DAY—AFTERNOON SESSION.

The Association was called to order at 2 P. M. by the president.

Dr. Richard Douglass, of Nashville, Tennessee, contributed a paper on

ABSCESS OF THE LIVER.

He said that abscess of this organ is the result of absorption of some morbid product from the intestines, or from some ulcerated surface. The bacteria enter the circulation and are deposited in the liver where abscess is formed. This may be with a normal temperature.

*Case.*—Four months after an attack of typhoid the patient had a chill, slight pyrexia, a trace of jaundice, this lasting only a few days. There was a globular swelling in the right hypochondriac region. The only symptoms were a dull heavy pain and tenderness. No general local disturbance. Diagnosis confirmed by aspiration. A free incision let out eight ounces of inodorous pus. Recovery in four weeks. In these cases the adhesions are diagnosed by palpation. When pus is detected it should be evacuated at once. Bleeding per aspiration needle or leeches applied to the abdomen he considers useful, although there is some risk attending aspiration.

Dr. G. W. Drake said that phagocytosis may furnish an explanation of the occurrence of abscesses in the various tissues and organs of the body, including the liver. The abscess may be a circumscribed locality in which was waged the hardest fought battles between the phagocytes and microbes, containing the disorganized remains of the slain, together with the products of decomposing tissue cells and microbes, under the dominion of chemical force, the flow of nerve force into the contents of the pus cavity being interrupted, or the nerve force transmuted into chemical force, if the doctrine of correlation of physical force may be extended to include nerve force.

Dr. E. E. Kerr, of Chattanooga, reported a case of Gall Stones.

Dr. J. B. Murfree, of Murfreesboro, Tennessee, read a paper on

UTERINE FIBROMA.

He said uterine fibroma is a morbid growth developed within the walls of the uterus, and is composed of muscular fibro-cells, fibro-plastic material and cellular tissue, and is due to a perversion of nutrition. It is non-malignant and homologous in its structure. Pain, hemorrhage, rectal and cystic irritations, indigestion, dropsy and exhaustion are some of its results. They threaten life by hemorrhage, inflammation, septicæmia and pressure.

The treatment is divided into four methods: (1) Symptomatic. (2) General, (by medicines). (3) Electrolysis. (4 Surgical.

By the first method we simply treat the symptoms as they arise and ward off threatened dangers. Hemorrhage is the most troublesome symptom and is best treated by quietude, opiates, etc. The hot douche and the tampon are useful.

The general treatment is by the administration of medicines to cause the absorption or destructiou of the tumors. Medicines are powerless to cause the absorption of a fibrous tumor and do no good, except to build up the general system. Ergot is given to cause the death and expulsion of the tumor. Dr. Murfree has no confidence in ergot for this purpose.

The treatment by electrolysis has met with some success and is worthy of trial. It is especially adapted to the interstitial variety.

Surgical treatment is most usually resorted to for the permanent relief of uterine fibroma. It consists in the removal of the tumor by traction, torsion, excision, enucleation, ecrassement and hysterectomy. When the tumor projects into the uterine cavity it is best removed by excision. When it is interstitial it should be treated by electricity. When subperitoneal it had better be let alone, unless the woman's life is a burden and death is threatening, when it should be removed through a laparotomy or by hysterectomy. Hysterectomy should never be resorted to as an ideal operation, but only a forlorn hope But conditions do arise when it is eminently proper and should unhesitatingly be performed.

Dr. Wm. H. Wathen, of Louisville, Kentucky, said the more frequently he goes into the pelvis the more often does he find that his diagnosis is not the same as it was before he had operated.

Apostoli's method he considered dangerous in private practice. To be successful one must be one of the most exclusive specialists. The tumor may be lessened in size, but there were, as far as he knew, no cures. A fibroma should not be interfered with unless it gives trouble.

Dr. Richard Douglas wished to emphasize what Dr. Wathen had said regarding Apostoli's treatment. From observation at his clinics and enquiries in the hospitals in Paris, he had not found any cases were cured, but he had heard of two cases that died as the result of the treatment, one of them being in the practice of Dr. Keith of London.

Dr. L. P. Barber, of Tracy City, Tennessee, said that Apostoli's method was certainly of great promise, notwithstanding the remarks of Drs. Douglas and Wathen. He saw something of the results of the treatment during the past summer, and he had the following results from his friend, Dr. Franklin H. Martin, of Chicago. Of 200 cases, three only had received no benefit. About fourteen per cent. received some benefit for a time, but this finally ceased. Eighty-four per cent. were symptomatically cured with a number of cases of actual cures. Dr. Martin has had no deaths in all his experience with electricity as compared with the results of hysterectomy and the use of the knife generally. The result to the unprejudiced, Dr. Barber thinks, is certainly in favor of the use of electricity in the right hands.

Dr. Murfree, in closing, said he felt that much could be accomplished by Apostoli's method.

Dr. Wm. H. Wathen followed with a paper entitled

### LAPAROTOMY VERSUS ELECTRICITY IN ECTOPIC PREGNANCY.

He said that electricity, the only feticidal means now recognized as orthodox by physicians who practice destroying the life of the fœtus in ectopic pregnancy without laparotomy, is no longer used for this purpose where the pregnancy has continued beyond three and a half or four months, and is seldom used after the third month. At this time the fœtus cannot be killed except by electro-puncture, and the complications and the deaths consequent upon this practice have been so numerous that the most radical advocates of electricity are afraid to introduce the electrodes into the gestation sac. The use of electricity in extra-uterine pregnancy is practically confined to the United States and while it is advocated by men of recognized ability and learning in obstetrics and gynecology, he was constrained to believe, that very soon it will have no support.

Dr. Wathen here entered into an argument in favor of laparotomy, for the difficulty and sometimes the impossibility of diagnosticating extra-uterine pregnancy in the early months is so manifest to experienced physicians, that it would be ridiculous to claim that in all these cases pregnancy existed ; while in the cases where laparotomy is done a diagnosis may positively be made by seeing the embryo or the chorionic or placental villi. If the embryo or fœtus in the extra-uterine pregnancy is killed by electricity, a more or less diseased condition of the pelvic structures is left that endangers the health or life of the woman; the dangers usually being increased as pregnancy advances. But if a laparotomy is done there is no obstructed tube, or other pathological condition left, and if the woman recovers from the immediate effects of the operation she is entirely cured. Her life is no longer in jeopardy because of the danger of pelvic abscess, sepsis or exhaustion following an effort to discharge the suppurating contents of the gestation sac through fistulous tracts in the rectum, vagina, bladder, or through the abdominal walls. If we could eliminate the cases where there was an error in diagnosis, we would find that the mortality from the use of electricity and the bad after results are far in excess of what follows laparotomy in the practice of experienced operators.

---

SECOND DAY—EVENING SESSION.

The Association met in Stone Church, and was called to order at 7:30 P. M.

An address of welcome was delivered by Dr. G. W. Drake, which was responded to by Dr. G. C. Savage, of Nashville.

Then followed the President's address, entitled

THE DOCTOR.

He said the man that starts out to be a doctor must understand that it is a life of toil. There is no flowery way to the royal degree of excellence. The laggard, the indolent, the careless never enter the temple of fame. The sluggard never tastes of the royal feast spread for the earnest, industrious worker. He knows nothing of the grandeur of mental comprehension of the real medical scholar. The doctor must be edu cated, not as that term seems to be understood in these days.

Modern education, Dr. Cowan feared, was too much a process of stuffing and cramming. The word education meant more than this. To educate was to draw out, to enlarge, to expand, to develop, and to strengthen.

---

OCTOBER 16TH—THIRD DAY—MORNING SESSION.

Dr. T. Hilliard Wood, of Nashville, Tennessee, contributed a paper on

HYPERTROPHY OF THE TONSILS, in which he said the treatment adopted for relief of hypertrophy of the tonsils had been subject to many variations. The treatment for the reduction of enlarged tonsils was divided into local, constitutional and operative. If the enlargement be due to swelling of the mucous membrane or to engorgement and congestion of the tonsil, the application of astringents may be of service. The most useful local remedies in the hands of the author are the sub-sulphate and perchloride of iron, about one to six or eight in water, or glycerine, and alum or tannin in powder. But where there is real overgrowth, the remedy, as Mackenzie well observes, must be of a destructive character, and escharotics, not astringents, must be used. Among escharotics, London paste is useful and should be applied once or twice a week. This will produce a slough, and repeated applications will reduce the gland to a normal size.

Constitutional measures to effect reduction of the tonsils include remedies to combat the diathesis upon which the enlargement often depends, iodide of potassium, cod liver oil and the general tonics, as the preparations of iron, and the bitter tonics.

With reference to operative treatment, excision by the tonsillotome is most popular, although the writer prefers the bistoury and vulsellum forceps. The operation is rendered painless by applying to the tonsil a solution of cocaine, and by injecting, with a hypodermic syringe, a few drops of the same solution into the substance of the gland. As a rule, general anæsthetics should not be used.

To reduce to a minimum the danger from hemorrhage, we have comparatively bloodless operations by the cold snare, ignipuncture, and the galvano-cautery amydalatome. Of these the galvano-cautery amydalatome seems preferable, and is highly recommended by Wright, of Brooklyn. Ignipuncture is tedious, requiring repeated applications, and attended by considerable pain. Moreover, it cannot be employed in the cases of refractory children.

Dr. N. C. Steele, of Chattanooga, said the amount of hemorrhage depended upon the condition of the tonsil. He would use the bistoury in adults, and the tonsillotome in children.

Dr. E. T. Camp has been able to reduce hypertrophied tonsils without operation, by using iodized phenol locally, and general remedies.

Dr. Geo. A. Baxter suggested painting the tonsil with flexible collodion.

Dr. Savage said there were two indications for operating upon the tonsils, viz., repeated attacks of tonsillitis and where breathing was interfered with. He uses only Mathews' tonsillotome. Never uses cocaine. From personal experience and observation he knew the operation was not very painful.

Dr. Gahagan suggested cold food in cases of incipient tonsillitis and plenty of cold liquids.

Dr. Reeves thinks in removing the tonsils we leave cicatricial tissue and so alter the voice. He uses tincture of iodine. He thinks cocaine will not control the hemorrhage.

Dr. Willis F. Westmoreland, of Atlanta, Georgia, said the frequency of enlarged tonsils was due to exposure, hence the greater number of cases observed in males. He would operate as soon as they gave trouble. He prefers the bistoury. Cocaine he has abandoned on account of increased bleeding. Ignipuncture was too painful.

Dr. Frank Trester Smith, of Chattanooga, resorts to ignipuncture where he cannot get consent to excision. Bleeding after the use of cocaine may continue for a long time. He said the indications for operating with him were interference with breathing, impairment of the voice and recurrent tonsillitis. The voice after operation improved as a rule.

THIRD DAY—AFTERNOON SESSION.

Dr. E. A. Cobleigh, of Chattanooga, read a paper entitled "A Case of Remarkable Injury with Recovery, and exhibited the patient. (Published on page 587.)

Dr. Willis F. Westmoreland, of Atlanta, Georgia, read a paper on

MORBID REFLEX NEUROSES AMENABLE TO SURGICAL TREATMENT.

Dr. H. Crumley, of Chattanooga, presented a case resembling epilepsy, which was examined by members of the Association.

Dr. J. R. Rathmell, of Chattanooga, reported a

CASE OF ABSCESS OF THE LIVER.

The patient had dysentery in July, 1889. Dr. Crumley was called to see the case December 17th. The abscess began to discharge through the lung December 21st and continued till February 27th, 1890, when he died. No autopsy.

Dr. Rathmell also reported a

CASE OF TYPHOID FEVER, and Dr. W. C. Townes, of Chattanooga, presented specimens of the intestines, spleen and mesentery of the case.

Dr. Townes then followed with a paper on

DILATED CARDIAC HYPERTROPHY, WITH NEPHRITIC COMPLICATIONS, illustrating his paper by specimens of the condition and others for comparison. Attention was called to the etiology, especially alcoholism, with its effects upon the kidney and liver. The specimens showed well the conditions mentioned and outlined. Heart's weight was 26½ ounces, normal heart being about nine ounces. The kidney was that termed by H. F. Formad, of Philadelphia, "pig-back."

In the treatment of this patient tincture of digitalis was administered; ten drops increased to sixty every hour. Then tincture of strophanthus was given, twenty drops, three times daily, with heart beats reduced from 124 per minute to 47.

THIRD DAY—EVENING SESSION

Dr. R. J. Trippe, of Chattanooga, reported a

CASE OF PERITONITIS, which occurred in a strong, muscular, heavy set negro, who had been struck with a crow-bar across the abdomen. The patient died.

Dr. C. H. Holland, of Chattanooga, reported a

CASE OF PHLEGMONOUS ABSCESS, occurring in an unusually large man, twenty-five years old, six feet high, and weight 349 pounds.

Dr. J. H. Atlee, of Chattanooga, followed with the report of a

CASE OF OVARIOTOMY.

Dr. Frank Trester Smith read a paper on

FLUORESCEIN IN THE DIAGNOSIS OF DISEASES OF THE EYE.

Dr. J. E. Purdon, of Cullman, Alabama, contributed a very elaborate paper on the

DYNAMICS OF MEDIUMISM, and, after a long and careful study of the subject, drew fifteen conclusions.

Dr. W. C. Maples, of Bellefonte, Alabama, read a paper on

IRREGULAR FORMS OF EPILEPSY, WITH REPORT OF A CASE.

He thinks his case was either one of epilepsy or hystero-epilepsy.

1. The amount of fever. In hystero-epilepsy there is generally but little or no fever. Some authors held that we may have a true hyterical fever, but the weight of authority is against that opinion.

2. The complete unconsciousness.

3. The biting of the tongue. Hystero-epileptics seldom or never injure themselves.

4. The facial expression and pupillary phenomena. The facial expression is generally calm and serene throughout a hystero-epileptic attack.

5. The absence of hysterial phenomena in the interval between the attacks.

6. The sex. While hystero-epilepsy does occur in males, all authors are agreed that it does so quite seldom.

The treatment was by large doses of bromide of potassium by the mouth, and morphine hypodermically. There were no sequelæ.

Dr. J. D. Gibson, of Birmingham, Alabama, presented a paper on

### URETHRAL STRICTURE AND ITS COMPLICATIONS.

In the use of sounds, he considers the spherical, or the acorn-pointed sound, the most convenient and practical instrument for the detection of stricture; while Otis and Weir and many others had their special instruments.

Dr. Gibson said that if he were compelled to use only one instrument in the cure of all strictures, he would use the sound. He believed that when it was properly used, that there were very few strictures that could not be relieved, and better so by it than any other means; and that the only stricture necessary to invoke the aid of the urethrotome was the old, tight and unyielding stricture in the pendent urethra and meatus. Young and inexperienced men were apt to be disappointed in the use of the sound, simply because they tried to go too fast; the idea should be to dilate the stricture and produce absorption, and not rupture.

Internal urethrotomy, while often abused, was a most potent means of treating urethral stricture, it being especially applicable for old and firm strictures in the pendent urethra and meatus.

Dr. P. S. Hayes, of Chicago, followed with a paper entitled

### NOTES ON APOSTOLI'S METHOD OF THE TREATMENT OF UTERINE FIBROIDS.

He said that one of the best demonstrated facts in the Apostoli operation was the arrest of all uterine hemorrhages, excepting those cases that are due to the puerperal condition. All observers unite in recognizing that the positive pole is the one to be connected with the intra-uterine electrode. To the thinking physician the query is, why the positive? And the answer comes that in electrolysis especially when the electrolyte—the fluid undergoing electrolysis—is blood, the clot formed around the positive pole is small and dense, while that around the negative pole is large and flabby. Knowing as we do that oxygen, chlorine and the acids are liberated at the positive pole when electrolysis is performed on the tissues of the body, and also knowing that hydrogen and alkalies are liberated around the negative pole, we have only to apply our knowledge of the action of the acids and alkalies respectively on the blood to explain the observed phenomena. The occurrence of uterine hemorrhage does not contraindicate the use of the method.

One of his patients suffering from menorrhagia came to his office, stating that she was drenched with the discharge and came for relief, as it was much easier for her to come to his office than to go home. The excessive flow did not occur until she had come down town. Dr. Hayes used the intra-uterine electrode connected with the positive pole, and allowed a current of from 60 to 80 milliamperes to pass for eight minutes. The patient then went home a distance of three miles and was n bed the remainder of the day. The next day she was about the house and the flow had nearly ceased. This period was by far the least severe she had had in several months, and the amount of time spent in bed was three-fourths less. The flow was diminished in like amount. Should opportunity again offer itself to use Apostoli's method during a non-puerperal hemorrhage, Dr. Hayes said he would not hesitate to use it as the best means of securing its arrest.

OFFICERS FOR 1891.

President—Dr. Robert Batty, Rome, Ga.

First Vice-President—Dr. E. T. Camp, Gadsden, Ala.

Second Vice-President—Dr. Richard Douglas, Nashville, Tenn.

Third Vice-President—Dr. D. H. Howell, Atlanta, Ga.

Secretary—Dr. Frank Trester Smith, Chattanooga, Tenn.

Treasurer—Dr. B. S. Wert, Chattanooga, Tenn.

On motion the Association adjourned to meet in Chattanooga, Tennessee (date to be decided on), 1891.

